# Integrated vector management for malaria control: a review of approaches and effectiveness

**DOI:** 10.1093/trstmh/traf084

**Published:** 2025-08-23

**Authors:** Gbeminiyi R Otolorin, María E Castellanos, Oyelola A Adegboye, Emma S McBryde

**Affiliations:** College of Medicine and Dentistry, James Cook University, Townsville, QLD 4811, Australia; Australian Institute of Tropical Health and Medicine, James Cook University, Townsville, QLD 4811, Australia; Centre for Tropical Biosecurity, James Cook University, Townsville, QLD 4811, Australia; Faculty of Veterinary Medicine, University of Jos, Jos, Plateau State 930001, Nigeria; College of Medicine and Dentistry, James Cook University, Townsville, QLD 4811, Australia; College of Medicine and Dentistry, James Cook University, Townsville, QLD 4811, Australia; Centre for Tropical Biosecurity, James Cook University, Townsville, QLD 4811, Australia; Menzies School of Health Research, Charles Darwin University, Darwin, NT 0811, Australia; College of Medicine and Dentistry, James Cook University, Townsville, QLD 4811, Australia; Australian Institute of Tropical Health and Medicine, James Cook University, Townsville, QLD 4811, Australia

**Keywords:** integrated vector management, IVM, malaria, malaria control, mosquitoes

## Abstract

Integrated vector management (IVM) is an effective strategy for reducing malaria transmission by combining various malaria vector control methods tailored to local contexts. The Web of Science, PubMed and Google/Google Scholar databases were used to gather studies related to IVM-based malaria control. This review synthesized findings from 14 studies published between 2009 and 2024 evaluating the impact of IVM on malaria control across different regions worldwide. The studies employed observational, quasi-experimental and cluster-randomized controlled trial designs, with outcome measures including malaria incidence, vector density, parasite prevalence, entomological inoculation rate and human biting rates. Integrated strategies consistently demonstrated greater effectiveness than single interventions, with six studies reporting statistically significant reductions in transmission (p<0.05) and several documenting notable declines in morbidity, mortality and entomological indicators. Longitudinal studies from Uganda, Ethiopia and Nigeria showed sustained reductions in malaria cases and vector populations, while large-scale programs in China and India illustrated the long-term success of coordinated, multisectoral IVM efforts. Emerging tools such as attractive targeted sugar baits, genetically modified mosquitoes and green-synthesized metallic nanoparticles offer more environmentally sustainable options. Combining traditional and innovative methods, IVM potentially provides a sustainable global malaria control and eradication solution.

## Introduction

Malaria is an important disease of global public health significance, contributing significantly to illness and death, particularly among children and pregnant women.^1,2^ As of 2021, nearly half of the world's population faced the threat of malaria infection, with the worldwide incidence of malaria estimated to be 247 million cases.^3^ In 2023, global malaria deaths were estimated at approximately 597 000, reflecting a slight reduction compared with the 608 000 fatalities recorded in 2022. The African Region remains disproportionately affected, accounting for >90% of global reported malaria infections and similarly >90% of all malaria-related mortalities.^3^

*Anopheles* mosquitoes are the sole known transmitters of malaria, with species such as *An. gambiae, An. stephensi, An. dirus, An. coluzzii, An. albimanus, An. funestus* and *An. a rabiensis* implicated. Hence, nearly all malaria vector control measures focus on preventing mosquito bites in humans.^4^ The main methods of vector control available in many malaria-endemic countries include insecticide-treated nets (ITNs), long-lasting insecticide-treated nets (LLINs) and indoor residual spraying (IRS), all of which use chemical insecticides.^2^ Alternative strategies target the larval or adult mosquitoes using biological control techniques or environmental management.^5^

Integrated vector management (IVM) employs a logical and evidence-based decision-making process to enhance the effectiveness of resources used in vector control efforts.^6^ These strategies are built on decision-making guided by reliable evidence, a combination of complementary vector control methods and strengthening community engagement and capacity.^6–9^ IVM aims to achieve effective, cost-efficient, environmentally sustainable and long-lasting vector control solutions.^10^

This mini-review evaluates the effectiveness of IVM strategies in controlling malaria by combining diverse approaches tailored to specific local needs. It highlights successful IVM applications in various regions of the world and evaluates the advantages of using innovative mosquito vector control tools approved or currently under evaluation by the World Health Organization (WHO), such as attractive toxic sugar baits (ATSBs, under evaluation), insecticide-treated wall liners (ITWLs; under evaluation), genetically modified mosquitoes (under evaluation), house screening (approved) and structural housing modifications (approved); these tools aim to further enhance the overall impact of IVM strategies.

## Literature search

### Search strategy

A review of published articles on IVM was conducted using the Web of Science, PubMed and Google Scholar/Google databases. The search applied the following criteria: ‘Integrated Vector Management’ OR ‘Integrated vector control’ OR ‘Integrated malaria vector control’ AND ‘Malaria’ AND ‘Mosquito’ (see Supplementary material SR1). The search was restricted to English-language publications. Articles were initially screened based on titles, followed by abstracts and full-text reviews.

### Inclusion and exclusion criteria

Studies were eligible for inclusion if they assessed the effectiveness of IVM interventions in malaria-endemic regions. Eligible interventions included combinations of malaria vector control strategies such as long-lasting insecticidal nets (LLINs), indoor residual spraying (IRS), larval source management (LSM; e.g. larviciding), environmental modification and community engagement. Studies were required to report at least one quantitative malaria-related outcome, including malaria incidence or prevalence, vector density, parasite prevalence, human biting rate (HBR) or entomological inoculation rate (EIR).

Studies were excluded if they evaluated only a single malaria vector control intervention without assessing its integration with other methods, lacked primary data, did not report measurable outcomes relevant to malaria transmission or entomological impact or were review articles, commentaries or editorials.

**Figure 1.**
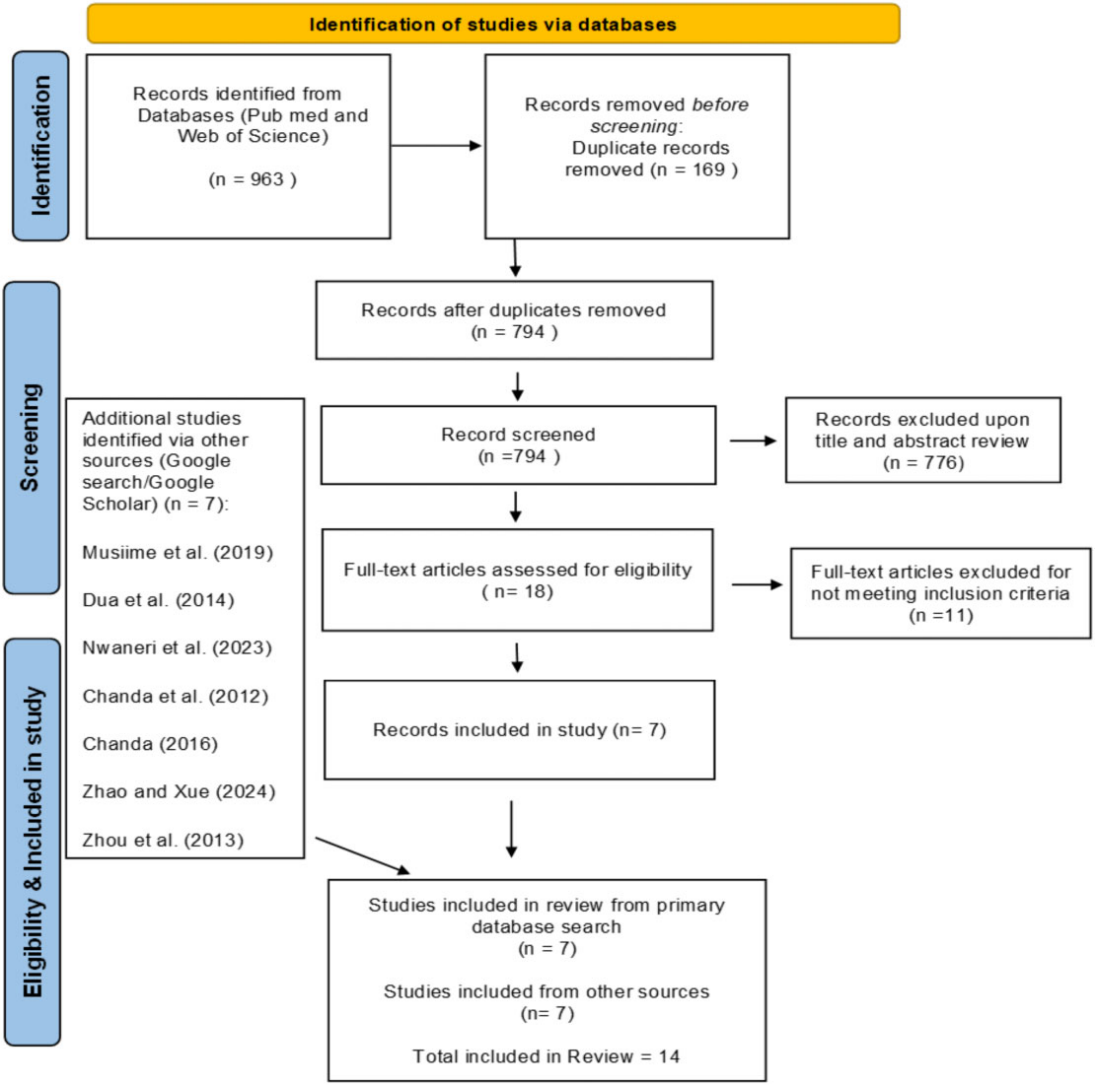
Study selection and search strategy flow chart.

After applying these eligibility criteria, 18 articles were identified for full-text review. Each article was thoroughly assessed for inclusion and studies that did not meet the criteria were excluded. This detailed screening process resulted in seven articles being selected for data collection and analysis. In addition to database searches conducted in Web of Science and PubMed, we identified a further seven eligible articles through targeted searches in Google and Google Scholar using predefined key terms. All studies from these additional sources were independently screened using the same inclusion criteria, which required the evaluation of malaria vector control interventions and the reporting of at least one quantitative malaria-related outcome. Eligible studies identified through these alternative sources were incorporated into the final review, resulting in a total of 14 articles included in the meta-analysis (Figure 1).

### Manuscript preparation

To ensure grammatical accuracy and clarity, the manuscript was proofread using AI-assisted Grammarly for Windows (https://www.grammarly.com).

## Results

### Study characteristics

The included studies were conducted between 1986 and 2021 across diverse malaria-endemic settings. They employed a range of methodological designs, including observational studies, quasi-experimental designs and cluster-randomized controlled trials. Each study evaluated the impact of IVM strategies on one or more malaria-related indicators. All 14 studies reported positive effects of IVM strategies on malaria control, with six studies demonstrating statistically significant reductions in malaria transmission (p<0.05). Table 1 provides an overview of the included studies, detailing the country of implementation, intervention strategies, study design, outcome measures and key findings. Figure 1 indicates the countries involved in this review.

**Table 1.** The impact of IVM, as reported in included studies.

Country (region)	IVM strategy	Measurement tested	Study type	Impact of IVM	Ref.
Kenya (Malindi and Nyabondo, western Kenya)	Biolarvicides, LLINs and IRS; collaboration and capacity building through advocacy	Malaria incidence	Observational study (mixed methods)	Malaria cases among children admitted to Malindi Hospital decreased from 23.7% (2006) to 10.47% (2011) (p<0.001)	^11^
Ethiopia (Botor-Tolay district, southwestern Ethiopia)	Community education and environmental management, larviciding, LLINs and IRS	Mosquito population (mosquito vector density), malaria incidence	Quasi-experimental pre-/postintervention	Adult mosquitoes collected decreased from 0.73/house/trap-night in 2015 to 0.37/house/trap-night in 2018 (p<0.001); a significant reduction in malaria cases occurred over 3 y, decreasing from 1162 in 2015 to 262 in 2018 (p<0.001)	^12^
Nigeria	LLINs and sanitation	Malaria incidence	Longitudinal community-based intervention study	Malaria incidence decreased from 16.8% in October 2017 to 1.3% in December 2017	^13^
Kenya and Ethiopia (Nyabondo plateau, Malindi subcounty and the Tolay region in southwestern Ethiopia)	Larviciding and LLINs, community education	Malaria prevalence	Factorial, cluster-randomized, controlled trial	Malaria prevalence in Tolay decreased by 50%, no significant malaria reduction in Malindi and Nyabondo	^14^
Tropical rainforest area of Cameroon	IRS and λ-cyhalothrin ITNs, enhanced house screening, outdoor misting	Mosquito infection rate	Quasi-experimental/pilot interventional study	Higher mosquito infection rate in non-treated villages; IRS+ITNs decreased transmission between May 2010 and April 2011	^15^
Kenya (Fort Ternan and Lunyerere	Environmental management (habitat modification, larviciding, *Gambusia affinis* fish)	Mosquito larval populations	Quasi-experimental community trial	Mosquito larvae numbers were higher in untreated habitats than treated ones (April 2008–March 2009)	^16^
Western Kenya	ITN and larviciding	Malaria incidence	Quasi-experimental design	Malaria incidence decreased from 60.4 per 1000 child-months to 34.5 (43% reduction) from November 2005 to January 2007	^17^
Uganda (15 districts)	Integrated vector control: larva source management, LLINs and IRS	Malaria incidence, malaria mortality	Longitudinal observational studies	Malaria infection rates decreased from 460 to 282 cases per 1000 people between 2013 and 2018; malaria mortality decreased from 320 deaths/day (2009) to <20/day (2018)	^18^
China	IRS, ITNs, irrigation management, rice–fish culture, public education	Malaria prevalence	Longitudinal observational study	Malaria was eliminated in 2021 after >70 years of IVM	^19^
Western Kenya	ITNs, IRS, *Bti*	Mosquito population reduction, malaria parasite prevalence reduction, malaria incidence reduction	Quasi-experimental cluster design	ITNs+IRS significantly reduced mosquito population (p<0.05); malaria parasite prevalence decreased by approximately 79% (p<0.05); malaria incidence decreased by 50% (p<0.05)	^20^
Uganda (Tororo District)	LLINs, IRS	Mosquito bites per person (HBR), infectious mosquito bites per person (EIR)	Longitudinal observational	The HBR showed a statistically significant decrease, from 19.6 to 2.3 bites per household per night (p<0.001); EIR decreased from 129 to 0 (p<0.001)	^21^
Hardwar, India	Source reduction, drainage improvement, bacterial larvicides, limited fogging	Malaria incidence	Longitudinal observational study spanning 25 y (1986–2011)	Malaria cases decreased from 2733 (1986) to 96 (1994) (p<0.001)	^22^
Brazil (Amazon)	ITNs and IRS	Mosquito bites per person (HBR) and malaria incidence rate	Longitudinal and cross-sectional studies	HBRs for *An. darlingi* decreased from 15.6 (first collection) to 4.0 (last collection). Malaria incidence rate decreased significantly, particularly for *Plasmodium vivax* and *Plasmodium falciparum*	^23^
Côte d'Ivoire (Northern Region)	LLIN+*Bti*	*Anopheles* spp. larval density	Randomized controlled trial	*Anopheles* spp. larval density: 0.61 (LLIN+*Bti*) vs 3.97 (LLIN only) (relative risk 6.50; p<0.001)	^24^

### Findings from studies

#### Types and description of IVM strategies

The reviewed studies employed a diverse set of IVM strategies, each designed to address malaria transmission through multipronged approaches adapted to specific ecological and epidemiological contexts. Among the interventions, the distribution and use of ITNs or LLINs was the most widely adopted, reported in 12 of 14 studies.^11–14,17–21,23,24^ The second most common was LSM, encompassing larviciding with microbial or chemical agents, habitat modification and source reduction, implemented in nine studies^11,12,14,16–18,20,22,24^ to target immature mosquito stages and reduce breeding sites. The third most used intervention was IRS, which was utilized in eight studies,^11,12,15,18–21,23^ serving also as an important measure to control adult mosquitoes.

Community engagement and health education were reported in six studies,^11,12,14,19^ often implemented through advocacy, participatory learning or public campaigns aimed at improving intervention uptake and behavioural compliance. Environmental management, such as improved drainage and the elimination of stagnant water bodies, was adopted in several studies.^12,15,16,22^ Biological control methods, including the use of *Gambusia affinis* fish and *Bacillus thuringiensis israelensis* (*Bti*) larvicides, were described in three studies.^16,20,24^ Ecological interventions such as irrigation management and rice–fish cultivation systems were featured in one study^19^ and structural measures like enhanced house screening and outdoor misting were reported in one study.^15^ Additionally, fogging was selectively employed in one study conducted within an industrial setting in India.^22^

The reviewed studies demonstrate a variety of IVM strategies combining multiple interventions to combat malaria across different regions. Commonly, LLINs and IRS were paired, as seen in Uganda,^21^ China^19^ and Brazil.^23^ In Kenya, combinations of LLINs, IRS and larviciding were implemented,^11^ while in Ethiopia, community education was integrated with LLINs and larviciding.^12^ Cameroon employed IRS, ITNs, enhanced house screening and outdoor misting.^15^ Environmental management strategies, which include habitat modification and larviciding, were used in malaria vector control in Kenya.^16^ In India, source reduction, drainage improvement, use of larvicides and limited fogging were combined.^22^

#### Efficacy of IVM in reducing malaria incidence and morbidity

Most of the included studies reported substantial reductions in malaria incidence following the implementation of IVM interventions. In Ethiopia, malaria cases declined significantly from 1162 in 2015 to 262 in 2018 (p<0.001), attributed to integrated measures including LLINs, IRS, larviciding and environmental management.^12^ In Nigeria, a rapid reduction in malaria prevalence was recorded, from 16.8% in October to 1.3% in December 2017, following a focused community-based IVM rollout.^13^ A quasi-experimental study in Kenya demonstrated a 43% decrease in child malaria incidence, from 60.4 to 34.5 per 1000 child-months over a 14-month period.^17^ Similarly, in western Kenya and Brazil, the combination of LLINs and IRS led to a significant reduction in malaria incidence (p<0.05).^20,23^

Beyond incidence, IVM strategies also significantly impacted malaria morbidity and mortality. In Uganda, a national IVM initiative involving LSM, LLINs and IRS resulted in malaria incidence decreasing from 460 to 282 cases per 1000 population between 2013 and 2018, alongside a dramatic reduction in malaria-related mortality, from 320 to <20 deaths per day.^18^ In Kenya (Malindi), a hospital-based assessment revealed a decrease in malaria-related admissions among children, from 23.7% in 2006 to 10.47% in 2011 (p<0.001), following an IVM program emphasizing capacity building and vector control.^11^ A long-term case from India illustrated the sustained success of IVM; within an industrial complex, malaria cases decreased from 2733 in 1986 to just 96 in 1994 (p<0.001).^23^ Furthermore, China achieved complete malaria elimination by 2021 after decades of coordinated IVM implementation.^19^

#### Efficacy of IVM in reducing malaria entomological indicators

In addition to reducing clinical outcomes, IVM strategies significantly impacted entomological indicators and key transmission intensity measures. For example, in Ethiopia, the adult mosquito density dropped from 0.73 to 0.37 mosquitoes per house/trap-night between 2015 and 2018 (p<0.001), demonstrating a substantial decrease in vector populations associated with larviciding and environmental management.^12^ The integration of LLINs and IRS in Uganda's Tororo District resulted in a dramatic decrease in rates of mosquito–human contact and effectively eliminated the potential for local malaria transmission, as evidenced by a statistically significant decrease in key entomological metrics (p<0.001).^21^ These changes reflect significant interruptions in the mosquito–human transmission cycle and are aligned with reductions in malaria incidence reported in the same settings.

Similar patterns were observed in additional studies. IVM strategies that combined LLINs with *Bti* larviciding significantly reduced mosquito populations and malaria transmission. In western Kenya, integrating ITNs, IRS and *Bti* larviciding led to substantial reductions in vector density, parasite prevalence (approximately 79%) and malaria incidence, all with statistical significance (p<0.05).^20^ Similarly, in Côte d’Ivoire, larval densities were markedly lower in LLIN and *Bti* areas than in LLIN-only areas (relative risk 6.50, p<0.001).^24^ In Kenya's highland villages, larval control measures were associated with notably lower mosquito densities in treated habitats compared with untreated ones, supporting the efficacy of environmental larval management.^16^ In Cameroon, higher sporozoite rates were observed in non-intervention villages, while IVM interventions (IRS and LLINs) contributed to a substantial transmission reduction over the course of a year.^15^

## Discussion

Malaria control has seen remarkable progress, with several countries successfully eliminating the disease through sustained and well-integrated interventions. The success stories of China, Azerbaijan, Belize and Tajikistan highlight the power of long-term commitment to malaria control, combining vector management, case detection and public health interventions.^19,25^ China's malaria elimination in 2021 highlights the effectiveness of IVM, where IRS, ITNs, irrigation management and public education were used in synergy.^26^ However, within sub-Saharan Africa, malaria transmission continues to be a persistent challenge due to favourable environmental conditions for mosquito breeding, increasing insecticide resistance and socio-economic barriers that limit access to preventive tools. Nonetheless, significant reductions in malaria incidence, parasite prevalence and mortality in countries like Uganda, Kenya, Cote d’Ivoire, Ethiopia and Nigeria indicate that IVM strategies, when consistently implemented, can reduce malaria transmission significantly. Uganda's sharp decline in malaria mortality, where daily deaths decreased significantly, is one of the most striking examples of IVM's success, reinforcing the need for sustained, comprehensive interventions tailored to local settings.^18^

One of the significant trends observed is that IVM is most effective when multiple control measures are integrated rather than relying on single interventions. While LLINs, IRS and larviciding are fundamental strategies for malaria prevention, they are not always effective on their own in regions where mosquitoes have developed resistance to insecticides or altered their behaviour to avoid these interventions. Studies in Kenya, Ethiopia and Uganda have shown that combining ITNs and IRS with larviciding and community-based environmental management has significantly reduced mosquito populations and malaria incidence. Nigeria's rapid 92% decrease in asymptomatic malaria infection within weeks of ITN distribution and improved sanitation demonstrates how simple interventions, when properly implemented, can yield fast and measurable results.^13^ Biological control methods, such as the use of *Bacillus*-based larvicides and predatory fish, have proven effective in reducing vector densities in regions like Ethiopia and western Kenya, particularly where resistance to synthetic insecticides has emerged. These combinations helped reduce HBRs and larval densities more effectively than single interventions, supporting their value in strengthening malaria control efforts. These findings suggest tailoring vector control strategies to local ecological and epidemiological contexts is crucial for maximizing impact.

As highlighted in this study, the two primary mosquito vector control methods, often deployed together or in combination with other complementary interventions, are ITNs/LLINs and IRS. Both tools have significantly contributed to reducing malaria transmission. However, their effectiveness is increasingly limited by practical and biological challenges. LLINs/ITNs are sometimes misused for non-health purposes, such as fishing, and access can be restricted for women and children in certain sociocultural settings. Furthermore, inadequate net maintenance and the failure to re-treat older nets reduce their protective efficacy.^27,28^ Notably, the growing emergence of insecticide resistance among malaria vectors, particularly pyrethroid resistance, poses a major threat to the effectiveness of both ITNs/LLINs and IRS.^29^ This resistance compromises the long-term success of these interventions and underscores the importance of continuous entomological surveillance and adaptive vector management strategies. These findings highlight the urgent need for complementary interventions, enhanced community engagement and the integration of innovative tools to sustain and strengthen the impact of malaria vector control programs.

Most studies relied on observational or quasi-experimental designs, offering practical insights into the effectiveness of IVM interventions. While longitudinal and quasi-experimental approaches have shown benefits and promising reductions in vector populations, especially in settings like Uganda, China and parts of East Africa, few fully randomized controlled trials have been conducted. This underscores the need for more rigorous experimental studies to strengthen the evidence base and assess the long-term impact of these interventions.

As mosquitoes continue to adapt, the response must evolve accordingly. A key emerging trend is the shift toward novel, eco-friendly control methods to supplement traditional interventions. ATSBs, which utilize sugar-based baits mixed with toxicants to target outdoor mosquitoes that are otherwise difficult to manage using conventional methods, are proving effective at targeting mosquitoes that evade indoor control measures like ITNs and IRS.^30^ Field trials in tropical regions have demonstrated the effectiveness of ATSBs in significantly reducing mosquito populations indoors and outdoors. By leveraging mosquitoes’ natural attraction to sugar, ATSBs provide a simple yet highly effective complement to existing control measures, particularly in regions where conventional methods have limited impact.^30^ While initial trials utilizing ATSBs show promise in reducing mosquito populations, recent epidemiological studies suggest a limited impact on decreasing malaria incidence.^31^ This discrepancy underscores the need for further investigation into the environmental and behavioural factors influencing ATSB effectiveness. Effective malaria control strategies should integrate ATSBs with other proven interventions and prioritize ongoing research to optimize their implementation across diverse settings. ATSBs may pose a risk to non-target sugar-feeding insects, including pollinators like bees and butterflies, especially if deployed outdoors. The use of mosquito-specific attractants and indoor or targeted applications can help reduce this risk.^32^

Similarly, gene drive technology and genetically modified mosquitoes, which employ CRISPR/Cas9 gene editing tools to either suppress mosquito populations or enhance their resistance to *Plasmodium* spp., are being explored as potential long-term, self-sustaining vector control strategies that could permanently disrupt malaria transmission cycles.^33^ Gene drive technology allow for the quick spread of genetic modifications through wild mosquito populations, significantly reducing malaria transmission. Combined with existing control methods, these genetic approaches offer a sustainable solution to insecticide resistance.^33^ With gene drive technology and CRISPR/Cas9-modified mosquitoes, the potential ecological concerns include disruption of food webs, loss of mosquito biodiversity and unintended gene flow to other species. Long-term effects on ecosystems are not yet fully understood, requiring robust risk assessments and containment strategies before widespread use.^34^ In Uganda (Tororo District), where IRS and ITNs significantly reduced HBRs and the EIR, these newer technologies could serve as additional safeguards to sustain gains and prevent resurgence.

Another major innovation is using green-synthesized metallic nanoparticles. These nanoparticles, derived from natural sources like plants, fungi, bacteria and insects, disrupt the mosquitoes’ biology, effectively controlling their populations. It offers an eco-friendly, non-toxic alternative to chemical insecticides.^35^ These advances align with a broader shift toward sustainability in malaria control, minimizing environmental impact while maintaining high efficacy. When incorporated into IVM strategies, nanoparticles provide a safer and more sustainable alternative to chemical insecticides, supporting long-term vector control.^35^ Green-synthesized metallic nanoparticles, although considered more eco-friendly than conventional synthetic chemicals, may accumulate in aquatic environments and potentially impact non-target organisms such as fish, algae and beneficial insects. Further research is needed to fully understand their long-term ecological safety.^36^

Plant-based solutions are also emerging as viable components of IVM strategies. Studies on plant extracts such as *Morinda citrifolia, Moringa oleifera* and *Ocimum basilicum* have highlighted their larvicidal and mosquito-repellent properties.^37^ These eco-friendly alternatives to chemical insecticides provide additional protection against malaria vectors.^37^ However, their misuse or unregulated application can have a negative impact on aquatic organisms, soil ecology and local plant biodiversity.

Another promising method is using ITWLs, which offer long-term protection by affixing insecticide-treated materials to the wall. Unlike IRS, which requires regular reapplication, this approach is cost-effective and sustained. Although insecticide resistance remains a concern, combining ITWLs with ITNs has improved malaria control outcomes.^38^ ITWLs pose minimal risk to non-target organisms due to indoor use. However, human exposure to insecticides and the potential for resistance development in mosquito populations are valid concerns.

Another innovative control method is the utilization of computer numerical control (CNC) knitting technology, which has led to the development of mosquito bite–blocking textiles. Designed with specialized knit patterns, these textiles provide effective protection against mosquito bites and can be integrated into IVM strategies as a wearable solution for personal protection.^39^ CNC-fabricated mosquito-blocking textiles pose negligible environmental risk, as they are purely mechanical barriers without chemical components. Environmental considerations focus on the sustainability of materials and energy consumption during production.

Eave Tubes (In2Care, Wageningen, The Netherlands) is another novel vector control tool that utilizes natural air currents to lure mosquitoes into ITN tubes installed in house eaves. This method has proven effective, particularly in regions with high insecticide resistance, complementing existing methods like ITNs and IRS for sustainable malaria control.^40^

House screening and structural housing modifications are increasingly recognized as effective, novel malaria vector control strategies that reduce human–mosquito contact by preventing mosquito entry and altering indoor resting sites. These interventions include the installation of insecticide-treated screens on windows, doors and eaves, sealing gaps, the use of Eave Tubes and structural upgrades like adding ceilings or plastering walls. By blocking or killing mosquitoes before they enter living spaces, these measures have demonstrated substantial efficacy, achieving 30–50% reductions in malaria incidence in multiple trials, particularly when integrated with standard tools like ITNs or IRS. For instance, a 2023 cluster-randomized trial in Côte d'Ivoire^41^ showed a 43% and 36% reduction in malaria infection prevalence measured by rapid diagnostic tests and microscopy, respectively, following implementation of a combined window screening and Eave Tube intervention. Beyond measured reductions in disease burden, recent studies also highlight strong community acceptance and perceived benefits: participants in Zambia^42^ reported reduced mosquito nuisance and malaria burden, while in Uganda, high-quality housing continued to confer protection against malaria even after adjusting for socio-economic status and ITN use.^43^ Additionally, Francisco^44^ noted that such modifications can enhance the reach of intervention by reducing dependence on consistent human behaviour (e.g. nightly net use). These findings underscore house screening and structural modifications as scientifically validated, community-supported and operationally sustainable tools that should be prioritized within IVM frameworks. These physical interventions have no known adverse environmental effects and are considered one of the most sustainable vector control methods.

Lastly, insecticidal paints have emerged as an innovative vector control tool under IVM. These paints incorporate insecticides into wall coatings where mosquitoes rest, providing long-lasting protection while minimizing the need for frequent reapplications. They offer an effective and sustainable solution, particularly in urban areas.^45^ Used indoors, these paints present low risk to non-target organisms, but human exposure to insecticides and resistance development must be monitored. Spills or improper disposal could affect soil or water if mismanaged.^46^

Integrating these innovations into national malaria control programs, securing appropriate funding and establishing robust regulatory frameworks are essential to maximizing their effectiveness. Ensuring safety, efficacy and sustainability in these vector control strategies will enhance the global response to malaria and contribute to its long-term elimination.^35^

Most studies in this review focused on Africa. This raises questions about how effectively these strategies might work in other regions with malaria. Future research should explore adapting and expanding these proven methods to suit different areas and circumstances. This involves ensuring that effective strategies are implemented and that new technologies are employed to enhance their effectiveness and sustainability. Ultimately, the goal of eliminating malaria relies on maintaining research, policy changes and community efforts working cohesively to stay ahead of the disease.

One of the key limitations of this review is the considerable heterogeneity in research methodologies, measured outcomes and geographic contexts across the included studies, which hinders direct comparison of results or consistent interpretation across studies. This review also does not fully address practical challenges of implementing integrated vector control, such as cost, logistics, long-term sustainability and the availability of trained personnel. In addition, many novel mosquito vector control methods such as gene drive technology, ATSBs, insecticidal paints and green-synthesized metallic nanoparticles are still in the early phases of research or limited field application. While a few studies have demonstrated promising results in reducing mosquito populations or vector contact, their overall impact on malaria transmission remains uncertain. Further large-scale, well-designed evaluations are needed to establish their effectiveness, environmental safety and suitability for integration into broader vector control programs.

The findings in this review show that strategies to combat malaria need to evolve as challenges like insecticide resistance and changes in mosquito behaviour persist. Countries that have successfully eradicated malaria stress the importance of long-term commitment, strong policies and enough funding. However, in places where malaria is still a big problem, there is a need for ongoing innovation and teamwork across different sectors.

Overall, these findings confirm that IVM is a highly effective and sustainable approach to malaria control, particularly when multiple interventions are integrated. The most successful interventions involved a combination of IRS, LLINs, larviciding and environmental management. Future malaria control efforts should focus on expanding large-scale trials, addressing insecticide resistance challenges and adapting IVM strategies to evolving vector behaviours to ensure long-term malaria control and elimination success.

## Conclusions

This review highlights the consistent effectiveness of IVM in reducing malaria transmission, vector density and related morbidity and mortality across diverse endemic settings. The included studies demonstrated that combining interventions such as LLINs, IRS, LSM and environmental control yielded greater impact than single-strategy approaches. IVM was shown to be adaptable to local contexts and capable of producing measurable improvements in both entomological and epidemiological outcomes. These findings reinforce IVM as a practical, evidence-based strategy for malaria control, particularly in high-burden regions. Future research should focus on optimizing combinations of interventions, evaluating long-term outcomes and scaling integrated strategies, while incorporating new innovative approaches to support sustained malaria reduction efforts.

## Supplementary Material

traf084_Supplemental_File

## Data Availability

No new data were generated during this study.

## References

[1] Kolawole EO, Ayeni ET, Abolade SA et al. Malaria endemicity in sub-Saharan Africa: past and present issues in public health. Microbes Infect Dis. 2023;4(1):242–51.

[2] Parra-Henao G, Coelho G, Escobar JP et al. Beyond the traditional vector control and the need to strengthen integrated vector management in Latin America. Ther Adv Infect Dis. 2021;8:2049936121997655. 33717481 10.1177/2049936121997655 PMC7922610

[3] World Health Organization . World malaria report 2024. Geneva: World Health Organization; 2024. Available from: https://www.who.int/teams/global-malaria-programme/reports/world-malaria-report-2024 [accessed].

[4] Takken W, Charlwood D, Lindsay SW. The behaviour of adult *Anopheles* gambiae, sub-Saharan Africa's principal malaria vector, and its relevance to malaria control: a review. Malar J. 2024;23:161. 38783348 10.1186/s12936-024-04982-3 PMC11112813

[5] Orok AB, Ajibaye O, Aina OO et al. Malaria interventions and control programs in Sub-Saharan Africa: a narrative review. Cogent Med. 2021;8(1):1940639.

[6] World Health Organization . Handbook for integrated vector management. Geneva: World Health Organization; 2012.

[7] Beier JC, Keating J, Githure JI et al. Integrated vector management for malaria control. Malar J. 2008;7(Suppl 1):S4. 19091038 10.1186/1475-2875-7-S1-S4 PMC2604879

[8] World Health Organization . Integrated vector management: working group meeting reports 2009. Geneva: World Health Organization; 2009.

[9] USAID . Integrated vector management programs for malaria vector control (version 2022) programmatic environmental assessment. Available from: https://www.icf.com/ [accessed 5 December 2024].

[10] Humblet MF, Losson B, Saegerman C. Integrated management of blood-feeding arthropods in veterinary teaching facilities – part 2: an overview of control methods against adults and immature stages. Rev Sci Tech. 2020;39(3):757–77. 35275138 10.20506/rst.39.3.3176

[11] Mutero CM, Mbogo C, Mwangangi J et al. An assessment of participatory integrated vector management for malaria control in Kenya. Environ Health Perspect. 2015;123(11):1145–51. 25859686 10.1289/ehp.1408748 PMC4629737

[12] Asale A, Kussa D, Girma M et al. Community-based integrated vector management for malaria control: lessons from three years experience (2016–2018) in Botor-Tolay district, southwestern Ethiopia. BMC Public Health. 2019;19:1318. 31638928 10.1186/s12889-019-7606-3 PMC6805624

[13] Nwaneri D, Ifebi E, Oviawe OO et al. Effects of integrated vector management in the control of malaria infection: an intervention study in a malaria endemic community in Nigeria. West Afr J Med. 2023;40(1):44–54. 36716288

[14] Mutero CM, Okoyo C, Girma M et al. Evaluating the impact of larviciding with *Bti* and community education and mobilization as supplementary integrated vector management interventions for malaria control in Kenya and Ethiopia. Malar J. 2020;19:390. 33143707 10.1186/s12936-020-03464-6 PMC7607826

[15] Matthews GA, Dobson HM, Nkot PB et al. Preliminary examination of integrated vector management in a tropical rainforest area of Cameroon. Trans R Soc Trop Med Hyg. 2009;103(11):1098–104. 19345969 10.1016/j.trstmh.2009.03.003

[16] Imbahale SS, Githeko A, Mukabana WR et al. Integrated mosquito larval source management reduces larval numbers in two highland villages in western Kenya. BMC Public Health. 2012;12:362. 22607227 10.1186/1471-2458-12-362 PMC3433356

[17] Fillinger U, Ndenga B, Githeko A et al. Integrated malaria vector control with microbial larvicides and insecticide-treated nets in western Kenya: a controlled trial. Bull World Health Org. 2009;87(9):655–65. 19784445 10.2471/BLT.08.055632 PMC2739910

[18] Chanda E . Exploiting the potential of integrated vector management for combating malaria in Africa. In: Rodriguez-Morales AJ, editor. Current topics in malaria. London: IntechOpen; 2016. Available from: https://www.intechopen.com/chapters/52580 [accessed].

[19] Zhao T, Xue R-D. Vector biology and integrated management of malaria vectors in China. Annu Rev Entomol. 2024;69:333–54. 38270986 10.1146/annurev-ento-021323-085255

[20] Zhou G, Lee MC, Githeko AK et al. Insecticide-treated net campaign and malaria transmission in western Kenya: 2003–2010. Malar J. 2013;12:1. 27574601 10.3389/fpubh.2016.00153 PMC4983699

[21] Musiime AK, Smith DL, Kilama M et al. Impact of indoor residual spraying and long-lasting insecticidal nets on malaria transmission in Uganda: a longitudinal cohort study. Malar J. 2019;18:76.30871535

[22] Dua VK, Pandey AC, Rai S et al. Impact of integrated vector management on malaria and its sustainability in an industrial complex in India. Am J Infect Dis. 2014;10(1):9–18.

[23] Martins-Campos KM, Pinheiro WD, Vítor-Silva S et al. Integrated vector management targeting *Anopheles darlingi* populations decreases malaria incidence in an unstable transmission area, in the rural Brazilian Amazon. Malar J. 2012;11:351. 23088224 10.1186/1475-2875-11-351 PMC3502175

[24] Tia J-PB, Tchicaya ESF, Zahouli JZB et al. Combined use of long-lasting insecticidal nets and *Bacillus thuringiensis israelensis* larviciding, a promising integrated approach against malaria transmission in northern Côte d'Ivoire. Malar J. 2024;23:168. 38812003 10.1186/s12936-024-04953-8 PMC11137964

[25] World Health Organization . Questions & answers on the world malaria report 2024. Geneva: World Health Organization; 2024. Available from: https://www.who.int/teams/global-malaria-programme/reports/world-malaria-report-2024/questions-and-answers [accessed 18 June 2025].

[26] World Health Organization . World malaria report 2023. Geneva: World Health Organization; 2023. Available from: https://www.who.int/teams/global-malaria-programme/reports/world-malaria-report-2023 [accessed 1 May 2023].

[27] Minakawa N, Dida GO, Sonye GO et al. Unforeseen misuses of bed nets in fishing villages along Lake Victoria. Malar J. 2008;7:165. 18752662 10.1186/1475-2875-7-165 PMC2532690

[28] Koenker H, Kilian A. Recalculating the net use gap: a multi-country comparison of ITN use versus ITN access. PLoS One. 2014;9(5):e97496. 24848768 10.1371/journal.pone.0097496 PMC4030003

[29] World Health Organization . Manual for monitoring insecticide resistance in mosquito vectors and selecting appropriate interventions. Geneva: World Health Organization; 2022. Available from: https://iris.who.int/bitstream/handle/10665/356964/9789240051089-eng.pdf?sequence=1 [accessed 2 March 2025].

[30] Njoroge TM, Hamid-Adiamoh M, Duman-Scheel M. Maximizing the potential of attractive targeted sugar baits (ATSBs) for integrated vector management. Insects. 2023;14(7):585. 37504591 10.3390/insects14070585 PMC10380652

[31] Ashton RA, Saili K, Chishya C et al. Efficacy of attractive targeted sugar bait stations against malaria in Western Province Zambia: epidemiological findings from a two-arm cluster randomized phase III trial. Malar J. 2024;23:343. 39548456 10.1186/s12936-024-05175-8 PMC11566550

[32] Diarra RA, Traore MM, Junnila A et al. Testing configurations of attractive toxic sugar bait (ATSB) stations in Mali, West Africa, for improving the control of malaria parasite transmission by vector mosquitoes and minimizing their effect on non-target insects. Malar J. 2021;20:184. 33853632 10.1186/s12936-021-03704-3 PMC8048058

[33] Kefi M, Cardoso-Jaime V, Saab SA et al. Curing mosquitoes with genetic approaches for malaria control. Trends Parasitol. 2024;40(6):492–503. 10.1016/j.pt.2024.04.010 38760256

[34] Jiang X, Wang W. Environmental release of gene drive systems: ecological risk assessment and monitoring framework development. GMO Biosaf Res. 2024;15(3):3837.

[35] Pramanik M, Roy P. Current prospects of green-metallic nanoparticles in mosquito control: a brief review. J Vector Borne Dis. 2025;62(2):143–53. 39113380 10.4103/JVBD.JVBD_17_24

[36] Ale A, Andrade VS, Gutierrez MF et al. Green-synthesized nanoparticles and their ecotoxicity in aquatic organisms. In: Desimone MF, Jotania RB, Khomane RB et al., editors. Nanotechnology. Cham: Springer; 2025:1–34.

[37] Opoku-Bamfoh O, Kwarteng SA, Owusu FAN et al. Repellent and larvicidal properties of selected indigenous plants in the control of *Anopheles* mosquitoes. J Vector Borne Dis. 2024;61:90–100. 38648410 10.4103/0972-9062.392267

[38] Mpangala KR, Halasa-Rappel YA, Mohamed MS et al. On the cost-effectiveness of insecticide-treated wall liner and indoor residual spraying as additions to insecticide-treated bed nets to prevent malaria: findings from cluster randomized trials in Tanzania. BMC Public Health. 2021;21:1666. 34521374 10.1186/s12889-021-11671-2 PMC8439046

[39] Holt B, Oswalt K, England A et al. Computer numerical control knitting of high-resolution mosquito bite-blocking textiles. Commun Eng. 2024;3:119. 39191889 10.1038/s44172-024-00268-3 PMC11350116

[40] Ni J, Wang J, Fang C et al. A review of the latest control strategies for mosquito-borne diseases. China CDC Wkly. 2024;6(33):852–6. 39211443 10.46234/ccdcw2024.183 PMC11350232

[41] Cook J, Sternberg ED, Aoura CJ et al. Housing modification for malaria control: impact of a “lethal house lure” intervention on malaria infection prevalence in a cluster randomised control trial in Côte d'Ivoire. BMC Med. 2023;21:168. 37143050 10.1186/s12916-023-02871-1 PMC10161487

[42] Saili K, De Jager C, Masaninga F et al. Community perceptions, acceptability, and the durability of house screening interventions against exposure to malaria vectors in Nyimba district, Zambia. BMC Public Health. 2024;24:285. 38267927 10.1186/s12889-024-17750-4 PMC10809574

[43] Nankabirwa JI, Gonahasa S, Katureebe A et al. The Uganda housing modification study – association between housing characteristics and malaria burden in a moderate to high transmission setting in Uganda. Malar J. 2024;23:223. 39080697 10.1186/s12936-024-05051-5 PMC11290271

[44] Francisco ME . House modifications for malaria control: enhancing protective coverage through structural interventions and community awareness. Trop Med Infect Dis. 2024;9:20.38251217

[45] Singh B, Kumar D, Kumar G et al. Insecticidal paint: an alternate integrated vector management strategy for mosquito control. Process Saf Environ. 2024;186:486–94.

[46] Schiøler KL, Alifrangis M, Kitron U et al. Insecticidal paints: a realistic approach to vector control? PLoS Negl Trop Dis. 2016;10(4):e0004518. 27101473 10.1371/journal.pntd.0004518 PMC4839634

