# Supplementary material for: Integrated vector management for malaria control: a review of approaches and effectiveness
Source: Trans R Soc Trop Med Hyg. 2025 Aug 23;119(11):1223–32. doi: 10.1093/trstmh/traf084 (PMC12580886; doi:10.1093/trstmh/traf084)
Supplement: traf084_Supplemental_File [file traf084_supplemental_file.docx]

**SUPPLEMENTARY MATERIALS**

**SR1.** Search Result.

1. Database -PUB MED( n=235)

Search string- "Integrated Vector Management" OR " Integrated vector control" OR " Integrated malaria vector control" AND "Malaria" AND "Mosquito".

1. Database- WEB OF SCIENCE (n=728)

Search string- "Integrated Vector Management" OR " Integrated vector control" OR " Integrated malaria vector control" AND "Malaria" AND "Mosquito"

1. Studies included from other sources(google search/google scholar) n = 7
   Musiime, et al (2019)

Dua, et al (2014))

Nwaneri, et al, 2023

Chanda et al 2012

Chanda, 2016

Zhao and Xue, 2024

Zhou et al, 2013
